# Assessing the metabolic effects of prednisolone in healthy volunteers using urine metabolic profiling

**DOI:** 10.1186/gm395

**Published:** 2012-11-30

**Authors:** Sandrine Ellero-Simatos, Ewa Szymańska, Ton Rullmann, Wim HA Dokter, Raymond Ramaker, Ruud Berger, Thijs MP van Iersel, Age K Smilde, Thomas Hankemeier, Wynand Alkema

**Affiliations:** 1 Division Analytical Biosciences, Leiden/Amsterdam Center for Drug Research, Einsteinweg 55, 2333CC Leiden, The Netherlands; 2 Netherlands Metabolomics Centre, Einsteinweg 55, 2333CC Leiden, The Netherlands; 3 MSD, Molenstraat 110, 5340 BH Oss, The Netherlands; 4 Biosystems Data Analysis, Swammerdam Institute for Life Sciences, University of Amsterdam, Amsterdam, The Netherlands; 5 TNO, Zeist, the Netherlands; 6 Synthon, Nijmegen, The Netherlands; 7 Department of Metabolic and Endocrine Diseases, University Medical Center Utrecht, Utrecht, The Netherlands; 8 Xendo, Groningen, The Netherlands; 9 PRA, Early Development Services, Zuidlaren, The Netherlands; 10 NIZO food research BV, Health Division, Ede, The Netherlands

**Keywords:** 3-methylhistidine, aminoaciduria, HOMA-IR, metabolomics, prednisolone, urine

## Abstract

**Background:**

Glucocorticoids, such as prednisolone, are widely used anti-inflammatory drugs, but therapy is hampered by a broad range of metabolic side effects including skeletal muscle wasting and insulin resistance. Therefore, development of improved synthetic glucocorticoids that display similar efficacy as prednisolone but reduced side effects is an active research area. For efficient development of such new drugs, *in vivo *biomarkers, which can predict glucocorticoid metabolic side effects in an early stage, are needed. In this study, we aim to provide the first description of the metabolic perturbations induced by acute and therapeutic treatments with prednisolone in humans using urine metabolomics, and to derive potential biomarkers for prednisolone-induced metabolic effects.

**Methods:**

A randomized, double blind, placebo-controlled trial consisting of two protocols was conducted in healthy men. In protocol 1, volunteers received placebo (*n *= 11) or prednisolone (7.5 mg (*n *= 11), 15 mg (*n *= 13) or 30 mg (*n *= 12)) orally once daily for 15 days. In protocol 2, volunteers (*n *= 6) received placebo at day 0 and 75 mg prednisolone at day 1. We collected 24 h urine and serum samples at baseline (day 0), after a single dose (day 1) and after prolonged treatment (day 15) and obtained mass-spectrometry-based urine and serum metabolic profiles.

**Results:**

At day 1, high-dose prednisolone treatment increased levels of 13 and 10 proteinogenic amino acids in urine and serum respectively, as well as levels of 3-methylhistidine, providing evidence for an early manifestation of glucocorticoid-induced muscle wasting. Prednisolone treatment also strongly increased urinary carnitine derivatives at day 1 but not at day 15, which might reflect adaptive mechanisms under prolonged treatment. Finally, urinary levels of proteinogenic amino acids at day 1 and of N-methylnicotinamide at day 15 significantly correlated with the homeostatic model assessment of insulin resistance and might represent biomarkers for prednisolone-induced insulin resistance.

**Conclusion:**

This study provides evidence that urinary metabolomics represents a noninvasive way of monitoring the effect of glucocorticoids on muscle protein catabolism after a single dose and can derive new biomarkers of glucocorticoid-induced insulin resistance. It might, therefore, help the development of improved synthetic glucocorticoids.

**Trial Registration:**

ClinicalTrials.gov NCT00971724

## Background

Glucocorticoids (GCs), such as prednisolone, represent the most important and frequently used class of anti-inflammatory drugs. Today, GCs are the standard therapy for reducing inflammation and immune activation in asthma, allergy, and inflammatory and autoimmune diseases, as well as in allotransplantation. In spite of excellent efficacy, the clinical use of GCs is hampered by a wide range of side effects, which are dependent on the administered dose and duration of treatment [1]. Persistent exposure to elevated levels of circulating GCs has been associated with metabolic derangements including the development of central adiposity, dyslipidemia, insulin resistance, glucose intolerance, diabetes and skeletal muscle wasting [1,2]. Both the anti-inflammatory and the metabolic effects of GCs are mediated through their binding to the GC receptor, which is ubiquitously expressed in the human body. Upon ligand binding, the GC receptor translocates into the nucleus where it enables initiation (transactivation) or suppression (transrepression) of target gene transcription. Whereas transrepression largely accounts for the anti-inflammatory action of GCs, transactivation of target genes involved in the metabolism of glucose, lipids or proteins is mostly implicated in adverse effects [3,4]. It has therefore long been hypothesized that it should be possible to design selective GC receptor agonists, with preserved transrepression actions and reduced transactivation effects, allowing the preservation of beneficial effects while reducing side effects [5,6]. However, development of selective GC receptor agonists has so far only resulted in a few compounds with improved therapeutic profiles in animal models [5,7], but proof of concept in human remains to be obtained. For evaluation and efficient development of such improved synthetic GCs, *in vivo *biomarkers, which can predict the occurrence of GC-induced side effects at an early stage, are highly desired.

In that respect, global metabolic profiling, or metabolomics, is an emerging technology that offers exciting promises. Metabolomics refers to the measurement of the metabolite pool that exists within a system under a particular set of conditions. It has been extensively applied to the area of drug research [8] and has proven useful for deriving early organ-specific biomarkers [9] as well as personalized medicine biomarkers that can be used to predict whether an individual will respond favorably or adversely to a drug [10]. Potential advantages of metabolomics over other omics platforms such as genomics, transcriptomics and proteomics is that metabolic changes may be more closely related to the immediate pathophysiologic state of an individual and that minimally invasive biofluids such as urine or blood are typically used.

To our knowledge, metabolomics has never been applied to study the effects of GCs in humans. Given the strong impact of these drugs on metabolism, we expect, however, that metabolomics is a valuable tool to derive early potential biomarkers for GC-induced metabolic effects. In the present study, we describe the untargeted mass spectrometry (MS)-based metabolomic analysis of urine samples from a clinical study in which healthy men were treated with increasing doses of prednisolone. In previous work on the same clinical trial, it was reported that prednisolone had induced various metabolic side effects in the volunteers, including insulin resistance [11]. The aims of the present work are to assess whether urine metabolomics can provide new insights into the dose-range and timeline of prednisolone-induced metabolic perturbations and to derive potential biomarkers of prednisolone-induced metabolic side effects

## Methods

### Patient treatment and sample collection

This study was single-centered, double blinded, randomized and placebo-controlled and consisted of two distinct parts. The two protocols enrolled healthy male volunteers (age range 20 to 45 years, body mass index 22 to 30 kg/m^2^) as described previously [11]. Briefly, health status and normal glucose metabolism were verified before enrolment, and volunteers were matched for age and body mass index between the treatment groups.

All participants provided written informed consent. This study was approved by the Stichting Beoordeling Ethiek Biomedisch Onderzoek and conducted in accordance with the Declaration of Helsinki using good clinical practice.

#### Protocol 1: two-week study

Placebo was administered to all volunteers (*n *= 47) on day 0 at 0800 h (baseline). The following day at 0800 h, participants were randomly assigned to a treatment with 7.5 mg (*n *= 11), 15 mg (*n *= 13) or 30 mg (*n *= 12) of prednisolone or with placebo (*n *= 11). Medication was taken once daily in the morning for a period of 15 days. This experimental setting will be referred as 'protocol 1' (Figure S1A in Additional file 1).

Urine samples were collected over 24 h on day 0, day 1 and day 15. No preservative was added to the urine samples. Fasting blood samples were collected in the morning of day 1, day 2 and day 16 prior to treatment. Samples were frozen at -80°C after collection. Fasting glucose and fasting insulin levels were measured and the homeostatic model assessment of insulin resistance (HOMA-IR) was calculated as previously described [11].

#### Protocol 2: acute study

The effects of acute treatment with prednisolone were assessed in different participants. Placebo was administered to all volunteers (*n *= 6) on day 0 at 0800 h (baseline). The following day at 0800 h, volunteers were treated with 75 mg prednisolone. This experimental setting will be referred as 'protocol 2' (Figure S1B in Additional file 1).

Urine samples were collected over 24 h on day 0 and day 1. No preservative was added to the urine samples. Fasting blood samples were collected in the morning of day 1 and day 2 prior to treatment. Samples were frozen at -80°C after collection.

### Metabolic profiling of urine samples

#### Sample preparation

Metabolomic analysis of urine samples was conducted by Metabolon, Inc. (Durham, NC, USA). Osmolality measurements were collected for each sample. Zirconia beads in a GenoGrinder (2 min, 675 spm (Glen Mills Inc., Clifton, NJ, USA)) were used to extract 100 μl of the urine samples in 400 µL ethyl acetate and ethanol (1:1). The sample was centrifuged and the liquid phase removed. The remaining pellet was re-extracted sequentially, with shaking, centrifugation and liquid recovery at each step, using 200 µL methanol, 200 µL methanol and water (3:1), and 200 µL dichloromethane and methanol (1:1). All resultant liquid phases were pooled (approximately 1 ml), then 225 µL aliquots were dried under a nitrogen stream in a Zymark TurboVap (Zymark, Runcorn, UK). The dried samples were then split into equal parts for analysis on the liquid chromatography- and gas chromatography-MS platforms as previously described [12].

#### Liquid chromatography-MS and gas chromatography-MS

For liquid chromatography-MS analysis, the dried extract was reconstituted in 100 μl 0.1% formic acid in 10% methanol. Liquid chromatography-MS was carried out using a Surveyor HPLC (Thermo-Electron Corporation, San Jose, CA, USA) with an electrospray ionization source coupled to a linear trap quadrupole mass spectrometer (Thermo-Electron Corporation), which consisted of an electrospray ionization source and linear ion-trap mass analyzer. Positive and negative ions were monitored within a single analysis alternating the ionization polarity of adjacent scans.

For gas chromatography-MS analysis, the dried extract was derivatized under dried nitrogen using bis(trimethylsilyl)trifluoroacetamide. The gas chromatography column was 5% phenyl and the temperature ramp was from 40°C to 300°C in a 16 min period. Samples were analyzed on a fast-scanning Thermo-Finnigan Trace DSQ Single Quadrupole mass spectrometer (ThermoElectron Corporation) using electron impact ionization. The instrument was tuned and calibrated daily for mass resolution and mass accuracy. More details on Metabolon's liquid chromatography-MS and gas chromatography-MS platforms can be found in [13]. Data are available upon request.

### Metabolic profiling of serum samples

Targeted metabolic profiling was conducted in serum samples prepared from fasting blood samples from volunteers of the placebo and 30 mg groups of protocol 1 and from all volunteers of protocol 2. Seventeen proteinogenic amino acids (alanine, arginine, asparagine, aspartic acid, glutamic acid, glutamine, histidine, isoleucine, leucine, lysine, phenylalanine, proline, serine, threonine, tryptophan, tyrosine, valine) and 3-methylhistidine were successfully measured in 5 μl of serum using a targeted liquid chromatography-MS/MS method adapted from [14]. Data are available upon request.

### Data analysis

#### Data pretreatment

To account for dilution effects between samples, each metabolite level in urine was normalized by osmolality measurement, which had a strong inverse correlation with total urine volume (R^2 ^= -0.87, *P *= 10^-49^, *n *= 153 samples). Prednisolone had no effect on total urine volume or osmolality at day 1. However, a significant increase in the total volume of urine excretion was observed at day 15 in all treatment groups (*P *= 0.03), which did not translate into a significant effect on osmolality (*P *= 0.08). Therefore, normalization to osmolality measurement was preferred to the more classical normalization to total urine volume. Missing ion intensity values were assumed to result from areas falling below the limits of detection. Metabolites with more than five missing values in one treatment group were discarded. For each remaining metabolite, the missing values were imputed with the observed minimum for that metabolite. In total, 515 peaks were measured in the urine samples, among which 177 metabolites were identified and used for subsequent analysis (listed in Table S1 n Additional file 2).

#### Principal component analysis

Principal component analysis (PCA) was performed using R [15] to assess the main sources of variation in metabolite composition of urine samples in volunteers from protocol 1. To improve visualization, the inter-individual variation in urinary metabolic composition was removed by subtracting metabolite concentration at baseline (day 0) from metabolite concentrations at day 1 and day 15. Three PCA models were fitted. The first one included metabolite levels in the urine of volunteers treated with placebo and 30 mg prednisolone only at day 1 and day 15, the second one included metabolite levels of volunteers treated with placebo, 7.5 mg, 15 mg or 30 mg prednisolone at day 1 only and the third one included metabolite levels of volunteers treated with placebo, 7.5 mg, 15 mg or 30 mg prednisolone at day 15 only. Metabolites were autoscaled prior to analysis.

#### Identification of metabolites significantly changed in protocol 1

To account for the paired structure of the data (more than one sample available for each individual), linear mixed models (LMMs) for repeated measurements were used to determine which metabolites were significantly changed in the urine of volunteers after treatment. LMMs were fitted using SAS (version 9.2, SAS Institute Inc., Cary, NC, USA), applying the method of residual maximum likelihood. For each metabolite, a separate model was built. It included metabolite concentrations of all individuals with specification of treatment group (placebo, 7.5 mg prednisolone, 15 mg prednisolone or 30 mg prednisolone), time (day 0, day 1, day 15) and individual (1, 2, ... 47). The outcome of the LMM was a global *P*-value of an F test with the H0 hypothesis that there is no difference between the mean metabolite concentrations of the eight time*treatment interaction groups. If this global *P*-value was lower than 0.05, then additional *t *tests were performed within the LMM output to test which pairs of time*treatment interaction groups were statistically significantly different. *P*-values of the latter *t *tests were tested for multiple comparisons including all metabolites using the false discovery rate [16] with a significance threshold q <0.05.

In serum, a similar data analysis strategy was used. One LMM was fitted for each metabolite as previously described for urine samples, using only placebo and 30 mg groups.

#### Identification of metabolites significantly changed in protocol 2

Because in protocol 2, no placebo group was included, paired *t *tests were conducted between metabolite concentrations at day 1 and metabolite concentrations at day 0, in urine and in serum. *P *<0.05 was considered as significant. *P*-values were tested for multiple comparisons including all metabolites using the false discovery rate with a significance threshold q <0.05.

#### Association between HOMA-IR and urine metabolites

To derive urinary metabolites that significantly correlated with HOMA-IR in protocol 1, partial least squares (PLS) regression analyses were applied using an in-house developed algorithm in MatLab version 7.9.0.529 R2009b (The MathWorks Inc., Natick, MA, USA) [17]. Two PLS models were built, regressing urinary MS data in all volunteers from protocol 1 (*n *= 47) as independent variables at day 1 or day 15 (X matrix) against HOMA-IR values at day 2 or day 16 (Y matrix) respectively. Metabolites were autoscaled prior to analysis. The statistical significance of the model performance and variable selection were assessed with 1,000 permutations of the Y matrix and *P *<0.05 was considered as significant

## Results

### Urinary metabolic profiles

#### Overview

**Figure 1:**
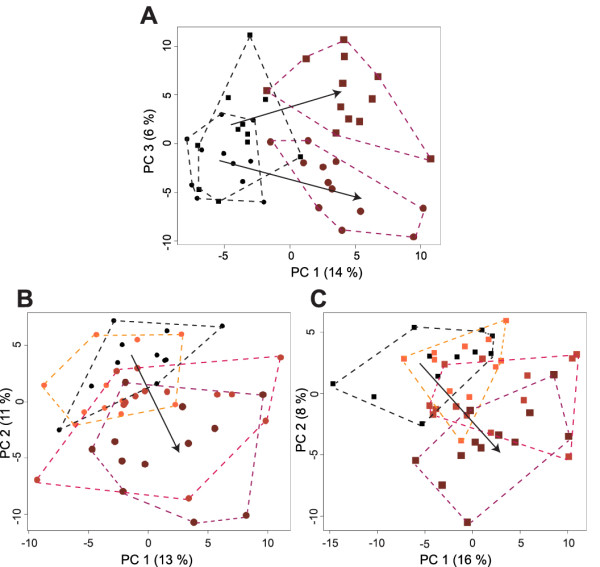
**PCA plots of urinary metabolic profiles**. **(A) **The first PCA model includes metabolic profiles from block 1 volunteers treated with placebo (black, *n *= 11) or 30 mg prednisolone (dark red, *n *= 12) for one day (circle) or 15 days (square). **(B) **The second PCA model includes metabolic profiles from block 1 volunteers treated with placebo (black, *n *= 11) or 7.5 mg (orange, *n *= 11), 15 mg (pink, *n *= 13) or 30 mg (dark red, *n *= 12) prednisolone for one day. **(C) **The third PCA model includes metabolic profiles from block 1 volunteers treated with placebo or prednisolone for 15 days. Arrows represent dose-dependent metabolic trajectories.

The dose- and time-dependent effects of prednisolone treatment were first assessed using protocol 1. In this protocol, 47 healthy men were treated with prednisolone (placebo (*n *= 11), 7.5 mg (*n *= 11), 15 mg (*n *= 13) or 30 mg (*n *= 12)) once daily for 15 days (Figure S1A in Additional file 1). Urine samples were collected at baseline, and after one day and 15 days of treatment and submitted to untargeted metabolic profiling. Figure 1 displays the overall results of the urine metabolic profiling. The first PCA model (Figure 1A) shows two diverging metabolic trajectories for the volunteers treated with 30 mg prednisolone at day 1 and day 15, whereas this effect was not seen in the placebo group. The other PCA models illustrate that these metabolic trajectories were strongly dose-dependent, after one day of treatment (Figure 1B), as well as after 15 days (Figure 1C). Prednisolone therefore induced both time- and dose-dependent metabolic perturbations in the urine of the volunteers.

#### Acute prednisolone treatment

After a single dose, prednisolone treatment significantly disrupted the levels of 31 metabolites in the urine of protocol 1 volunteers. In the volunteers treated with 7.5 mg, 15 mg and 30 mg prednisolone, 2, 10 and 29 metabolites were significantly changed, respectively, compared with placebo (Table 1). This confirmed that the effects of prednisolone on the urinary metabolic profiles of healthy volunteers were dose-dependent. Prednisolone treatment decreased the urinary levels of dehydroepiandrosterone sulfate (DHEA-S), and strongly increased the levels of glucose and of metabolites involved in lipid metabolism such as propionylcarnitine, L-acetylcarnitine and L-carnitine. Prednisolone treatment also consistently dose-dependently increased the urinary levels of 13 proteinogenic amino acids: lysine, alanine, histidine, methionine, threonine, proline, serine, leucine, valine, phenylalanine, glycine, asparagine and isoleucine.

**Table 1:** Metabolites significantly changed in urine of healthy volunteers treated with prednisolone for one day.

Metabolite			Protocol 1				Protocol 2
pathway	Name	HMDB ID	Placebo	7.5 mg	15 mg	30 mg	75 mg
Lipid metabolism	L-carnitine	HMDB00062	1.4	1.9	2.2	9.0***	8.4***
	Propionylcarnitine	HMDB00824	1.4	3.0	2.9**	5.2***	5***
	L-acetylcarnitine	HMDB00201	1.5	2.4	2.8**	7.1***	5.9***
	Myoinositol	HMDB00211	0.9	1.1	1.6	3.2***	4.5***
Proteinogenic amino acids	L-lysine	HMDB00182	0.8	1.0	1.6	2.1***	2.1***
	L-alanine	HMDB00161	1.1	1.1	1.3*	1.6***	1.9***
	L-histidine	HMDB00177	1.0	1.1	1.5	2.4***	1.9**
	L-methionine	HMDB00696	1.1	1.3	1.2	1.6***	1.8***
	L-threonine	HMDB00167	1.0	1.0	1.2	1.6***	1.8**
	L-proline	HMDB00162	1.0	1.2	1.2	1.7***	1.7**
	L-serine	HMDB00187	1.0	1.1	1.2	1.5***	1.7**
	L-leucine	HMDB00687	1.0	1.2	1.0	1.4***	1.6*
	L-valine	HMDB00883	1.0	1.1	1.1	1.4***	1.5***
	L-phenylalanine	HMDB00159	1.0	1.2	1.1	1.3***	1.4***
	Glycine	HMDB00123	1.0	1.0	1.2	1.4**	1.4**
	L-asparagine	HMDB00168	0.8	0.9	1.0	1.2***	1.5
	L-isoleucine	HMDB00172	1.1	1.1	1.0	1.6**	1.4
Other amino acids	4-guanidinobutanoate	HMDB03464	1.2	1.4	1.8	2.8***	4.0***
	Beta-alanine	HMDB0056	1.0	1.4*	1.7*	2.2***	2.8*
	Pyroglutamine	NA	1.3	1.4	1.4	1.9**	1.9*
	L-kynurenine	HMDB00684	1.1	1.1	1.6	1.4**	1.3*
Carbohydrate metabolism	D-glucose	HMDB00122	1.2	1.4	1.5	8.9***	16.7*
	3-hydroxybutyrate	HMDB00023	1.0	1.3	1.4*	2.0***	2.8**
Steroids	Dehydroepiandrosterone sulfate	HMDB01032	1.3	0.8	0.8*	0.8*	0.4**
Energy metabolism	Phosphate	HMDB01429	1.2	1.3	0.9**	0.9**	0.9
Nucleotide metabolism	Nicotinic acid mononucleotide	HMDB01132	1.2	1.4	1.5**	1.3	1.4**
	Nicotinamide	HMDB01406	1.5	1.5	1.0**	1.0**	1.4
	1-methylguanosine	HMDB01563	1.0	1.2*	1.1	1.0	1.0
Vitamins and cofactors	Pantothenate	HMDB00210	1.1	1.3	1.3*	1.3**	1.4***
	L-dehydroascorbate	HMDB01264	1.1	1.2	1.1	1.2**	1.2
Xenobiotics	Mandelic acid	HMDB00703	1.0	0.9	1.1	1.4**	0.9

Data represents the mean ratio of metabolite level at day 1 compared with day 0. *P <0.05, **P <0.01,***P <0.001 and q <0.05 compared to placebo group for protocol 1 using liner mixed models, and compared to day 0 for protocol 2, using paired *t *tests. HMDB, Human Metabolome Database.

**Figure 2:**
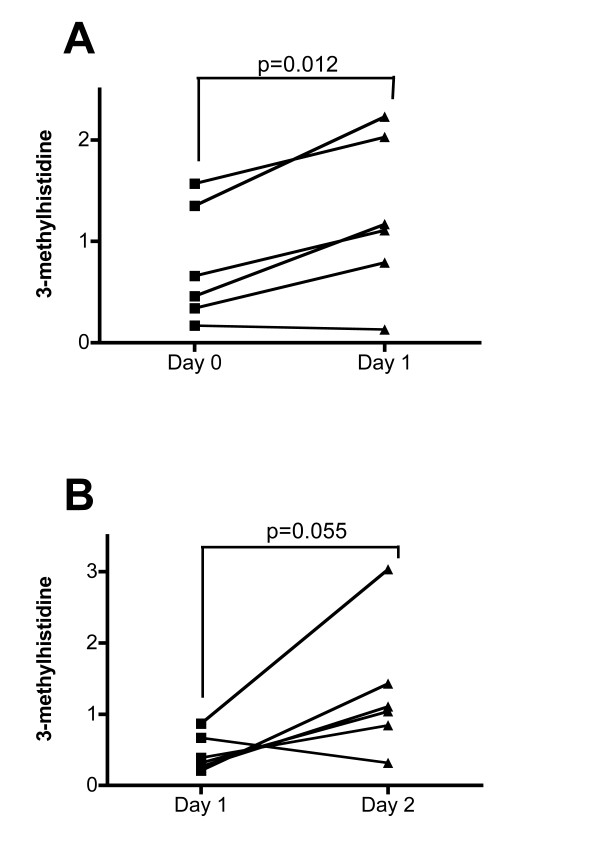
**3-methylhistidine in protocol 2 volunteers**. Data represent metabolite levels (divided by the mean of 3-methylhistidine level detected in this study) in urine **(A) **and serum **(B) **of protocol 2 volunteers before and after a single dose of prednisolone (75 mg). *P*-values calculated using paired *t *tests.

To evaluate the robustness of these findings in an independent cohort, we analyzed the urine samples from protocol 2 volunteers using the same metabolomic platforms. In this protocol, six independent healthy men were treated with placebo at day 0 and with 75 mg prednisolone at day 1 and urine samples were collected at both time-points (Figure S1B in Additional file 1). Among the 31 metabolites that were selected in protocol 1, 24 were similarly significantly changed in protocol 2 (Table 1). Therefore, the rapid effects of prednisolone on urinary amino acids, glucose, DHEA-S and carnitine derivatives were confirmed. Interestingly, we also observed that, among many others, 3-methylhistidine, a marker for muscle protein catabolism, was significantly increased in this protocol (Figure 2A). A list of all urinary metabolites significantly changed during this study, including protocol 2, is provided in Table S2 in Additional file 3.

#### Prolonged prednisolone treatment

To assess the difference between a single dose and a longer therapeutic treatment, the same analysis was conducted on the urinary metabolic profiles of protocol 1 volunteers at day 15. At this time-point, 29 metabolites were significantly changed (Table 2). Similar to day 1, the effects of prednisolone on urinary metabolic profiles were dose-dependent. It is noteworthy that, for some metabolites, a strong effect was seen in the urine profiles of volunteers from the placebo group, which made detection of prednisolone-specific effects less reliable (see for example metabolites involved in catecholamine metabolism in Table 2). Therefore, later discussion in this paper will focus only on metabolites for which the effect in the placebo group was small or prednisolone-specific effects were confirmed in several dose groups, time-points or matrices. Thus, after 15 days, the effects of prednisolone on urine glucose and DHEA-S levels observed at day 1 were still present and levels of seven proteinogenic amino acids (glutamine, histidine, asparagine, threonine, tryptophan, serine and isoleucine) were still elevated. By contrast, carnitine derivatives returned to baseline levels (Table S2 in Additional file 3).

**Table 2:** Metabolites significantly changed in urine of healthy volunteers treated with prednisolone for 15 days.

Metabolite pathway			Protocol 1			
	Name	HMDB ID	Placebo	7.5 mg	15 mg	30 mg
Proteogenic amino acids	L-glutamine	HMDB00641	0.9	1.5**	4.1*	3.3***
	L-histidine	HMDB00177	1.2	0.8	1.3	2.3**
	L-asparagine	HMDB00168	0.9	1.1**	1.3**	1.6***
	L-alanine	HMDB00161	1.0	1.0	1.3	1.6***
	L-threonine	HMDB00167	0.9	0.9	1.0*	1.4***
	L-tryptophan	HMDB00929	1.0	1.1	1.1	1.3***
	L-serine	HMDB00187	1.0	0.9	1.0	1.2*
	L-isoleucine	HMDB00172	1.2	0.9	0.9**	1.3
Other amino acids	Kynurenate	HMDB00715	1.1	0.9	0.8***	0.9**
	Prolyl-4-hydroxyproline	HMDB06695	1.1	1.1	0.8**	0.8**
Carbohydrate metabolism	D-glucose	HMDB00122	1.2	1.4	1.4	5.5*
	Gluconate	HMDB00625	1.3	1.1	1.0**	0.9***
	N-acetylgalactosamine-4-sulfate	HMDB00781	1.2	1.0	0.9	0.8***
	Sorbitol	HMDB00247	1.2	1.0	0.9*	0.8*
Steroids	Dehydroepiandrosterone sulfate	HMDB01032	0.9	0.2***	0.2**	0.2***
Energy metabolism	Isocitrate	HMDB00193	1.1	1.1	0.9*	0.8***
	Phosphate	HMDB01429	1.1	1.0	0.8*	0.8**
	Citrate	HMDB00094	1.4	1.0*	1.0**	0.7***
Nucleotide metabolism	Nicotinic acid mononucleotide	HMDB01132	1.4	1.3	1.0*	0.9*
	Pseudouridine	HMDB00767	1.1	1.0	1.0	0.9*
	N-methylnicotinamide	HMDB00699	1.2	0.9	0.6*	0.5**
Vitamins and cofactors	Pyridoxine	HMDB00239	1.2	0.7**	0.7	0.8**
Xenobiotics	Mandelic acid	HMDB00703	1.0	1.1	1.0	1.4**
	Salicyluric acid	HMDB00840	2.5	1.3	1.0**	1.0
	Tartaric acid	HMDB00956	1.1	1.1	0.9*	0.9
Catecholamine metabolism	Homovanillic acid	HMDB00118	1.2	0.9*	0.9**	1.0*
	Dopamine	HMDB00073	1.3	0.9**	0.8***	0.9**
	Vanillylmandelic acid	HMDB00291	1.1	1.0	0.9	0.9*
Glycerophospholipid metabolism	Ethanolamine	HMDB00149	1.0	0.8**	0.8**	0.9*

Data represents the mean ratio of metabolite level at day 15 compared to day 0. *P <0.05, **P <0.01,***P <0.001 and q <0.05 compared to placebo group, using linear mixed models. HMDB, Human Metabolome Database.

### Targeted metabolomics in serum

To exclude kidney failure as the cause of prednisolone-induced aminoaciduria, we measured proteinogenic amino acids in serum samples from the volunteers treated with the highest doses of prednisolone (30 mg and 75 mg) (Table 3).

**Table 3:** Proteinogenic amino acids in serum of healthy volunteers treated with prednisolone.

Metabolite		Day 2			Day 16	
Name	HMDB ID	Placebo	30 mg	75 mg	Placebo	30 mg
L-glutamine	HMDB00641	1.1	1.4	1.6***	1.5	1.4
L-alanine	HMDB00161	1.1	1.3**	1.5***	1.2	1.3
L-asparagine	HMDB00168	1.2	1.3	1.5**	1.2	1.2
L-arginine	HMDB00517	1.3	1.0	1.4*	1.5	1.1
L-aspartic acid	HMDB00191	1.2	1.1	1.4*	1.3	1.1
L-phenylalanine	HMDB00159	1.1	1.1	1.2**	1.1	1.0
L-proline	HMDB00162	1.1	1.1	1.2**	1.2	1.2
L-threonine	HMDB00167	1.1	1.1	1.2**	1.1	1.0
L-tyrosine	HMDB00158	1.2	1.1	1.2*	1.1	1.0
L-tryptophan	HMDB00929	1.1	1.2	1.1*	1.2	1.1
L-serine	HMDB00187	1.2	1.1	1.3	1.2	1.0
L-histidine	HMDB00177	1.1	1.0	1.1	1.1	0.9
L-isoleucine	HMDB00172	1.1	1.1	1.1	1.0	0.9
L-lysine	HMDB00182	1.2	1.0	1.1	1.1	1.0
L-glutamic acid	HMDB00148	1.1	0.9	1.0	1.1	1.0
L-leucine	HMDB00687	1.1	1.0	1.0	1.0	0.8
L-valine	HMDB00883	1.1	0.9	0.9	1.0	0.9

Data represents the mean ratio of metabolite level at day 2 or day 16 compared with day 1. *P <0.05, **P <0.01,***P <0.001 and q <0.05 compared to placebo group for protocol 1 using liner mixed models, and compared to day 0 for protocol 2, using paired *t *tests. HMDB, Human Metabolome Database.

After one day of treatment, one proteinogenic amino acid (alanine) significantly increased in the serum of the volunteers treated with 30 mg prednisolone compared with placebo, and 10 (glutamine, alanine, asparagine, arginine, aspartic acid, phenylalanine, proline, threonine, tyrosine and tryptophan) increased in the serum of the volunteers treated with 75 mg prednisolone compared with baseline levels. After 15 days of treatment, no significant change in amino acid concentration was observed in the serum of volunteers treated with 30 mg prednisolone compared to placebo.

Because, at least in the highest dose group (75 mg), kidney failure could then be excluded as the cause of prednisolone-induced aminoaciduria, levels of 3-methylhistidine, a marker for muscle protein catabolism, were also investigated in serum of the same volunteers. In block 1, no significant difference was seen between 30 mg and placebo groups (data not shown), whereas in block 2 volunteers, levels of 3-methylhistidine were increased (*P *= 0.055) (Figure 2B).

### Urinary biomarkers for prednisolone-induced insulin resistance

#### HOMA-IR

**Figure 3:**
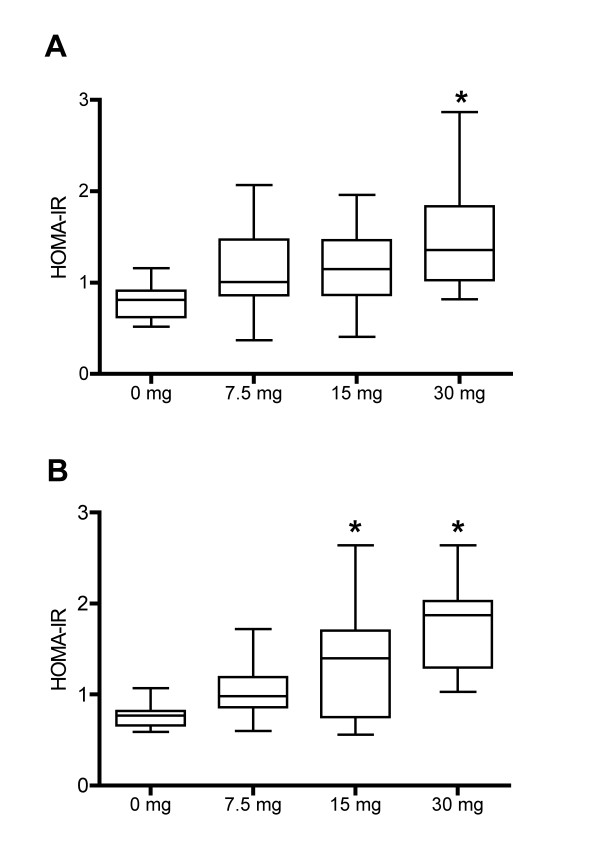
**HOMA-IR n volunteers from protocol 1**. **(A) **Day 2. **(B) **Day 16. The black lines represent the mean value. The top and bottom of the box represent the 75th and 25th percentile. The whiskers indicate the maximum and minimum points. *P <0.05 compared to placebo group, using analysis of variance.

Finally, we aimed to evaluate the relationship between the urinary metabolic perturbations induced by prednisolone and the development of insulin resistance. HOMA-IR, an index for measuring insulin resistance, was calculated in volunteers from protocol 1 at day 2 and at day 16 (Figure 3). HOMA-IR was not perturbed by 7.5 mg prednisolone at any time-point; 15 mg prednisolone increased HOMA-IR after 15 days; and 30 mg prednisolone increased HOMA-IR at day 2 and day 16. Thus, prednisolone treatment enhanced insulin resistance in healthy volunteers in a dose- and time-dependent manner.

#### Partial least squares regressions

Two PLS regression models were constructed between metabolite levels in urine and HOMA-IR separately at day 1 and at day 15 (Table 4). Urinary metabolic profiles and HOMA-IR were significantly correlated at both time-points (*P *= 0.015 and *P *= 0.004 respectively). At day 1, 10 proteinogenic amino acids and two carnitine derivatives positively correlated to HOMA-IR. At day 15, proline betaine, tartaric acid, androsterone sulfate, N-methylnicotinamide (NMN), pimelic acid, isocitric acid and taurine negatively correlated with HOMA-IR, whereas L-alanine and N-acetylvaline positively correlated with HOMA-IR.

**Table 4:** Summary of partial least squares regressions between urinary metabolic profiles and HOMA-IR.

Day	*P*	Metabolite name	HMDB ID	R	*P*
Day 1	0.015	L-proline	HMDB00162	+	0.002
		L-alanine	HMDB00161	+	0.003
		L-glutamic acid	HMDB00148	+	0.006
		L-valine	HMDB00883	+	0.006
		L-leucine	HMDB00687	+	0.011
		L-serine	HMDB00187	+	0.017
		Propionylcarnitine	HMDB00824	+	0.022
		L-phenylalanine	HMDB00159	+	0.024
		L-isoleucine	HMDB00172	+	0.029
		L-methionine	HMDB00696	+	0.032
		L-acetylcarnitine	HMDB00201	+	0.044
		L-histidine	HMDB00177	+	0.045
Day 15	0.004	Tartaric acid	HMDB00956	-	0.013
		Androsterone sulfate	HMDB02759	-	0.013
		Proline betaine	HMDB04827	-	0.014
		N-methylnicotinamide	HMDB03152	-	0.016
		Pimelic acid	HMDB00857	-	0.025
		L-alanine	HMDB00161	+	0.027
		N-acetylvaline	HMDB11757	+	0.028
		Taurine	HMDB00251	-	0.037
		Isocitric acid	HMDB00193	-	0.05

The sign of the correlation coefficients to the regression axe (R) and *P*-values are displayed only for metabolites significantly associated with HOMA-IR. HMDB, Human Metabolome Database, HOMA-IR, homeostatic model assessment of insulin resistance.

## Discussion

The present study first aimed at providing an unbiased description of the metabolic perturbations induced by prednisolone treatment in human using untargeted urine metabolic profiling. Development of GC-adverse metabolic effects has mainly been studied with high doses (30 to 60 mg) [18,19]. However, most patients treated with prednisolone for long periods receive doses lower than 7.5 mg per day and the extent to which these low doses induce metabolic adverse effects is still unclear. In a recent study, it has been shown that 7.5 mg prednisolone daily for 15 days affects multiple pathways of intermediary metabolism in healthy volunteers [20], however the observed perturbations were rather small. In the present study, we provide evidence that both the acute and prolonged effects of prednisolone on human metabolism are strongly dose-dependent. At the lowest therapeutic dose (7.5 mg), changes in metabolic profiles were indeed almost nonexistent, even after 15 days of treatment, whereas in the highest doses (30 mg and 75 mg), urine metabolic profiles were strongly perturbed after a single dose.

Moreover, we demonstrate differential metabolic effects of acute versus prolonged treatment with similar doses of prednisolone. PCA highlighted two diverging urinary metabolic trajectories at day 1 and day 15, especially in the highest dose groups. These findings were confirmed by more refined statistical analyses, which highlighted different metabolic pathways for the metabolites disrupted by prednisolone treatment at day 1 and at day 15. Previous studies have shown differential metabolic effects of single versus repeated dose of GCs. Based on data obtained from the same clinical trial, Van Raalte *et al*. reported that an acute high dose of prednisolone inhibited insulin secretion in healthy volunteers, whereas insulin secretion increased at day 15, illustrating that β-cell function partially recovered following prolonged exposure [11]. In light of these previous findings, our new results might also reflect adaptive mechanisms triggered in healthy volunteers under prolonged treatment with prednisolone.

One of the most important finding of this study was that prednisolone increased the urinary concentrations of 13 proteinogenic amino acid after a single dose. In healthy humans, amino acids are usually almost fully reabsorbed in the kidney proximal tubules and very low quantities are excreted in urine. Aminoaciduria only occurs if kidney transport is affected or if plasmatic concentrations increase [21]. Here, aminoaciduria cannot be attributed to impaired renal amino acid reabsorption, because protein amino acid levels were also increased in serum of the volunteers treated with the highest dose of prednisolone at day 1. Moreover, GCs have previously been shown to enhance kidney amino acid reabsorption in rats [22,23].

It is well described that high doses of GCs acutely induce protein catabolism in healthy young adults [24-26]. Therefore, the simultaneous increase of urine and serum proteinogenic amino acids in volunteers treated with high doses of prednisolone observed in our study at day 1 might reflect this catabolic effect of prednisolone. Burt *et al*. reported that prednisolone-stimulated protein oxidation does not persist under chronic administration and that a metabolic adaptation occurs to limit protein loss [27]. In our study, prednisolone-induced aminoaciduria only partially persisted after 15 days. A smaller number of amino acids was indeed significantly increased at day 15 compared to day 1 (7 versus 13 in the 30 mg group), which suggests partial metabolic adaptation in the healthy volunteers.

Prolonged exposure to GCs is frequently associated with marked skeletal muscle atrophy [28] resulting from decreased protein synthesis and increased protein breakdown [29,30]. In the volunteers of protocol 2, we observed increased urinary and serum levels of 3-methylhistidine, an amino acid formed by the methylation of certain histidine residues in the myofibrillar proteins actin and myosin. In humans, 3-methylhistidine cannot be reused for muscle protein synthesis when these proteins are broken down [31] and is not metabolized but rapidly excreted unchanged in urine [32]. Moreover, because 90% of the body pool of 3-methylhistidine resides in skeletal muscle [32] and this tissue contributes as much as 75% to urinary 3-methylhistidine [33], it has been proposed that the measurement of urinary excretion of this amino acid could be used to assess the rate of breakdown of skeletal muscle protein [34]. In the present study, rapid increase of 3-methylhistidine therefore supports the hypothesis that prednisolone-induced aminoaciduria is an early manifestation of the well-known GC-induced skeletal muscle atrophy. However, the fraction of 3-methylhistidine excretion that can be attributed to skeletal muscle may vary depending on circumstances, and other sources of 3-methylhistidine have been reported [35,36]. To measure more quantitatively the contribution of prednisolone-induced skeletal muscle breakdown in healthy individuals, more invasive measurements such as muscle interstitial 3-methylhistidine concentrations [37] could be performed.

In the clinic, the use of prednisolone in doses of lower than 10 mg/day is rarely associated with GC-induced myopathy, whereas higher GC doses result in a more rapid onset of muscle weakness [38]. Our results are in agreement with these clinical observations, because we did not observe any disruption in the urinary amino acid profiles of healthy volunteers treated with 7.5 mg/day and the first significant increase in amino acid levels was observed at 15 mg/day.

Overall, we found that one of the earliest effects of prednisolone on the metabolism of healthy volunteers involves amino acid metabolism and that it is most likely an early manifestation of GC-induced skeletal muscle wasting. We therefore suggest that urinary metabolomics represents a noninvasive way of monitoring the effect of GCs on protein catabolism as soon as after a single dose.

In addition to the effects on amino acids, prednisolone significantly disrupted the levels of many other urinary metabolites. For example, we observed that prednisolone strongly increased the urinary levels of carnitine, acetyl-L-carnitine and propionylcarnitine at day 1 but not at day 15. These three endogenous compounds are part of the total carnitine pool. Because more than 90% of the total body store of carnitine resides within skeletal muscle [39], these observations could best be explained by the myopathic phenotype induced by prednisolone resulting in the loss of these three metabolites from the muscle tissue and subsequent urinary excretion. The fact that the carnitine derivative levels return to normal after 15 days could be part of the metabolic adaptation mentioned earlier. However, the normal physiological role of these metabolites is linked with the oxidation of fatty acids, and their decreased urinary levels have been shown to be potent biomarkers for the activation of β-oxidation [40]. Here, a temporary inhibition of fatty acid oxidation might also be induced by prednisolone in healthy volunteers. We cannot rule out the possibility of prednisolone-induced alteration of carnitine renal reabsorption. Additional studies are needed to unravel the possible cause of this transient prednisolone-induced increase in carnitine derivatives.

We also describe that DHEA-S levels strongly decreased in the urine of volunteers at day 1 and day 15, even at the lowest dose (7.5 mg) of prednisolone. DHEA-S is a major metabolite of DHEA. They are both mainly produced by the adrenal cortex, and over 99% of DHEA is sulfated before secretion. Because of its long half-life compared with DHEA, circulating DHEA-S levels serve as a measure of integrated adrenal androgen secretion. It is well described that exogenous GC administration has a profound impact on both DHEA and DHEA-S production [41]. Therefore, our present findings are not surprising. However, we show here that urine metabolomics could already follow adrenal androgen suppression after a single low dose of prednisolone, as well as its progression over time, as DHEA-S levels were lower after 15 days than after one day compared with placebo. Interestingly, decreased DHEA levels have been implicated in high cholesterol, inflammation, immune disorders, diabetes and osteoporosis [42,43], and DHEA replacement has attracted considerable attention over recent years [44]. However, whether the prednisolone-induced reduction of DHEA and DHEA-S levels contributes to prednisolones undesired side effects remains unknown.

Finally, we demonstrate that the urinary metabolic profiles of healthy individuals treated with prednisolone are significantly correlated to HOMA-IR, a clinical measure of insulin resistance. Interestingly, metabolites that correlate to HOMA-IR after a single dose or after 15 days of prednisolone treatment are different, suggesting that different metabolic pathways are involved when insulin resistance is induced by a single high dose of prednisolone treatment or by repeated treatment.

After one day of prednisolone treatment, urinary levels of 10 amino acids and two short chain acyl-carnitines were positively associated with HOMA-IR. Similar metabolic profiles of altered protein and branched-chain amino acid metabolism have been associated with insulin resistance in men [45]. Therefore, perturbation of branched-chain amino acid metabolism is thought to be an important component in the development of insulin resistance and our results suggest that this might also be the case with regards to GC-induced insulin resistance after acute treatment.

After 15 days, NMN was the only metabolite that was both significantly correlated with HOMA-IR and significantly decreased by treatment. NMN is a metabolite of nicotinamide, which is itself a precursor of nicotinamide adenine dinucleotide. In human and rodent urine, it was previously found that 'species demonstrated profound changes in nucleotide metabolism, including that of NMN [...], which may provide unique biomarkers for following type 2 diabetes progression' [46]. In diabetic individuals, decreased urinary levels and slower plasma clearance of NMN after nicotinamide overload have been observed [47,48]. Nicotinic acid and nicotinamide have been reported to induce insulin resistance [49,50] and NMN is thought to trigger this effect. In light of these previous findings and our results, NMN might therefore represent an interesting biomarker for prednisolone-induced insulin resistance.

## Conclusions

MS-based metabolomics detected time- and dose-dependent changes in the urine of healthy volunteers treated with prednisolone. After one single dose, a strong aminoaciduria probably reflected GC-induced muscle protein catabolism in the highest dose groups. Metabolic perturbations differed between acute and prolonged treatment, suggesting adaptive mechanisms. Finally, some urinary metabolites were significantly associated with HOMA-IR, including branched-chain amino acids and NMN, which might represent interesting biomarkers of prednisolone-induced insulin resistance. This study illustrates that the application of metabolic profiling can improve our understanding of GC-induced metabolic side effects, provide early biomarkers for them with minimally invasive procedures and therefore help the development of improved synthetic GCs. It might also prove useful in the future to monitor, and ultimately predict, the appearance of GC-induced side effects on an individual basis.

## Abbreviations

DHEA: dehydroepiandrosterone; DHEA-S: dehydroepiandrosterone sulfate; GC: glucocorticoid; HOMA-IR: homeostatic model assessment of insulin resistance; LMM: linear mixed model; MS: mass spectrometry; NMN: N-methylnicotinamide; PCA: principal component analysis; PLS: partial least squares.

## Competing interests

SES, ES, RR, RB, AKS and TH declare that they have no competing interests. TR, WHAD, TMPI and WA were previously employed by MSD.

## Authors' contributions

SES contributed to experiments, analyzed the data and wrote the manuscript; ES and AKS analyzed the data and revised the manuscript; TR, WHAD and WA conceived the project and wrote/revised the manuscript; RR measured proteinogenic amino acids in the serum samples; RB and TH contributed to data interpretation and revised the manuscript. TMPI supervised that clinical trial. All authors have read and approved this manuscript for publication.

## Additional data files

The following additional data are available with the online version of this paper. Additional data file 1 is a figure illustrating the experimental design. Additional data file 2 is a table listing all metabolites measured in urine samples. Additional data file 3 is a table listing metabolites significantly changed in urine of volunteers of protocols 1 and 2.

## Supplementary Material

Additional file 1 **Figure S1: Illustration of the experimental design**. **(A) **Protocol 1. On day 0 at 0800 h, placebo was administered to 47 healthy men. On day 1 at 0800 h, volunteers were randomly assigned to a treatment with 7.5 mg (*n *= 11), 15 mg (*n *= 13) or 30 mg (*n *= 12) of prednisolone or with placebo (*n *= 11). Medication was taken once daily in the morning for 15 days. A 24 h urine sample was collected at day 0, day 1 and day 15 and fasting blood samples were collected in the morning of day 1, day 2 and day 16 prior to treatment. **(B) **Protocol 2. On day 0 at 0800 h, placebo was administrated to six healthy men. On day 1 at 0800 h, volunteers were treated with 75 mg prednisolone. A 24 h urine sample was collected at day 0 and day 1. Fasting blood samples were collected in the morning of day 1 and day 2 prior to treatment. Click here for file

Additional file 2 **Table S1: List of all metabolites detected and identified in urine of healthy volunteers**. Click here for file

Additional file 3 Table S2: List of all metabolites significantly regulated in urine of healthy volunteers treated with prednisolone, in protocol 1 and 2. Data represents the mean ratio of metabolite level at day 1 or day 15 compared to day 0. *P <0.05, **P <0.01,***P <0.001 and q <0.05 compared to placebo group for protocol 1 using LMMs, and compared to day 0 for protocol 2, using paired *t *tests. Click here for file
