# Supplementary figures and images for: Assessing the metabolic effects of prednisolone in healthy volunteers using urine metabolic profiling
Source: Genome Med. 2012 Nov 30;4(11):94. doi: 10.1186/gm395 (PMC4064315; doi:10.1186/gm395)

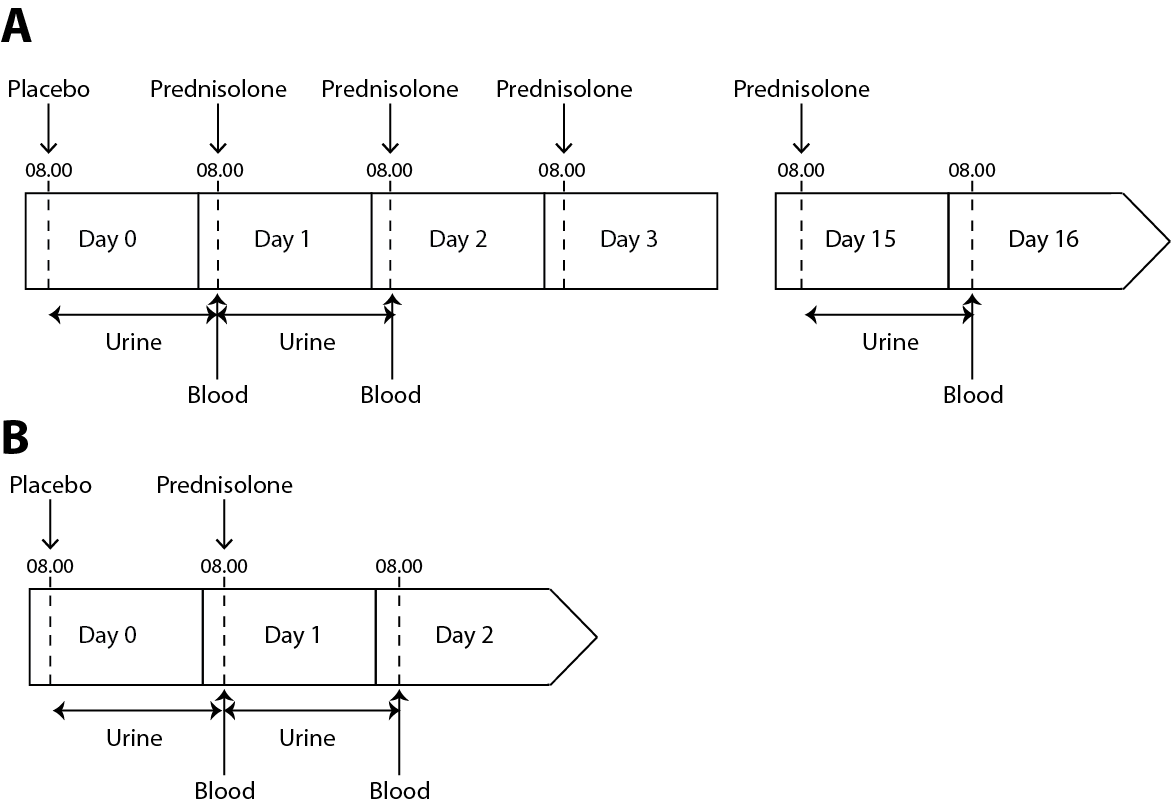

Supplement: Additional file 1 — Figure S1: Illustration of the experimental design. (A) Protocol 1. On day 0 at 0800 h, placebo was administered to 47 healthy men. On day 1 at 0800 h, volunteers were randomly assigned to a treatment with 7.5 mg (n = 11), 15 mg (n = 13) or 30 mg (n = 12) of prednisolone or with placebo (n = 11). Medication was taken once daily in the morning for 15 days. A 24 h urine sample was collected at day 0, day 1 and day 15 and fasting blood samples were collected in the morning of day 1, day 2 and day 16 prior to treatment. (B) Protocol 2. On day 0 at 0800 h, placebo was administrated to six healthy men. On day 1 at 0800 h, volunteers were treated with 75 mg prednisolone. A 24 h urine sample was collected at day 0 and day 1. Fasting blood samples were collected in the morning of day 1 and day 2 prior to treatment. [file gm395-S1.PNG]
