# Supplementary material for: Assessing the metabolic effects of prednisolone in healthy volunteers using urine metabolic profiling
Source: Genome Med. 2012 Nov 30;4(11):94. doi: 10.1186/gm395 (PMC4064315; doi:10.1186/gm395)
Supplement: Additional file 2 — Table S1: List of all metabolites detected and identified in urine of healthy volunteers. [file gm395-S2.PDF]

Supplemental Table 1: List of all metabolites detected and identified in urine of healthy volunteers.

Metabolite	HMDB ID	PubChem ID
(S)-3-Hydroxyisobutyric acid	HMDB00023	87
(S)-b-aminoisobutyric acid	HMDB02166	439434
1-Methylguanidine	HMDB01522	10111
1-Methylguanosine	HMDB01563	250902
1-Methylhistidine	HMDB00001	92105
1-Methyluric acid	HMDB03099	69726
2-Hydroxyglutarate	NA	NA
2-Hydroxyphenethylamine	HMDB01065	1000
2,3-Dihydroxyvaleric acid	HMDB00421	20848966
3-Alpha-mannobiose	NA	419524
3-Hexenedioic acid	HMDB00393	107550
3-Hydroxyisovaleric acid	HMDB00754	69362
3-Hydroxymethylglutaric acid	HMDB00355	1662
3-Hydroxyphenylacetic acid	HMDB00440	12122
3-Methylhistidine	HMDB00479	64969
3,4-Dihydroxybenzeneacetic acid	HMDB01336	547
4-Acetamidobutyric acid	HMDB03681	18189
4-Guanidinobutanoic acid	HMDB03464	500
4-Hydroxybenzoic acid	HMDB00500	135
4-Hydroxyestrone	HMDB05895	18418
4-Pyridoxic acid	HMDB00017	6723
4-Ureido-butyrate	NA	NA
5-Hydroxyindoleacetic acid	HMDB00763	1826
5-Methyl-deoxycytidine monophosphate	NA	NA
5-Methyluridine	HMDB00884	445408
5-Sulfosalicylic acid	HMDB11725	7322
5'-Methylthioadenosine	HMDB01173	149
Acetylphosphate	HMDB01494	186
Adenine	HMDB00034	190
Adenosine	HMDB00050	60961
Adenosine 3,5-cyclic monophosphate	HMDB00058	6076
Agmatine	HMDB01432	199
Allantoin	HMDB00462	204
Alpha-Hydroxyisobutyric acid	HMDB00729	4277439
Alpha-N-Phenylacetyl-L-glutamine	HMDB06344	92258
Androsterone sulfate	HMDB02759	159663
Arabic acid	NA	10264
Ascorbic acid	HMDB00044	644104
Azelaic acid	HMDB00784	2266
Beta-Alanine	HMDB00056	239
Biopterin	HMDB00468	2380
Cellobiose	HMDB00055	439178
Choline	HMDB00097	305
cis-Aconitic acid	HMDB00072	309
Citramalic acid	HMDB00426	441696
Citric acid	HMDB00094	311
Citrulline	HMDB00904	9750
Cl adduct of formate dimer	NA	NA

Metabolite	HMDB ID	PubChem ID
Cotinine	HMDB01046	408
Creatine	HMDB00064	586
Creatinine	HMDB00562	588
D-Fructose	HMDB00660	439709
D-Galactose	HMDB00143	439357
D-Glucose	HMDB00122	5793
D-Glucuronic acid	HMDB00127	444791
D-Mannose	HMDB00169	18950
D-Xylitol	HMDB02917	6912
D-xylonate	NA	6602431
D-Xylulose	HMDB01644	619
Dehydroisoandrosterone 3-sulfate	HMDB01032	12594
Diaminopimelic acid	HMDB01370	865
Dihydrofolic acid	HMDB01056	98792
Dihydrouracil	HMDB00076	649
Dopamine	HMDB00073	681
Erythritol	HMDB02994	8998
Erythronic acid	HMDB00613	439535
Estriol	HMDB00153	5756
Ethanolamine	HMDB00149	700
Fumaric acid	HMDB00134	723
Glucaric acid	HMDB00663	33037
Gluconic acid	HMDB00625	10690
Glucosamine	HMDB01514	439213
Glutamyl-valine	NA	NA
gly-leu, acetyl-lys, ala-val	NA	NA
Glycerol	HMDB00131	753
Glycerol 3-phosphate	HMDB00126	439162
Glycine	HMDB00123	750
Glycolic acid	HMDB00115	757
Glycylproline	HMDB00721	79101
Guanidoacetic acid	HMDB00128	763
Guanine	HMDB00132	764
Hexanoyl-DL-carnitine	HMDB00756	6426853
Hippuric acid	HMDB00714	464
Homocysteine	HMDB00742	778
Homovanillic acid	HMDB00118	1738
Hydroxyphenyllactic acid	HMDB00755	9548580
Hypoxanthine	HMDB00157	790
Indole-3-lactic acid	HMDB00671	92904
Indoleacetic acid	HMDB00197	802
Indoxyl sulfate	HMDB00682	17530
Isocitric acid	HMDB00193	1198
Kynurenic acid	HMDB00715	3845
L-Acetylcarnitine	HMDB00201	18230
L-alanine	HMDB00161	5950
L-Arabinose	HMDB00646	439195
L-Asparagine	HMDB00168	6267
L-Aspartyl-L-phenylalanine	HMDB00706	93078
L-Beta-imidazolelactate	NA	NA
L-Carnitine	HMDB00062	10917
L-Cysteine	HMDB00574	5862

Metabolite	HMDB ID	PubChem ID
L-Dehydroascorbate	HMDB01264	210328
L-Gamma-glutamyl-L-leucine	HMDB11171	4524287
L-Glutamic acid	HMDB00148	33032
L-Glutamine	HMDB00641	5961
L-Gulonolactone	HMDB03466	439373
L-Histidine	HMDB00177	6274
L-Isoleucine	HMDB00172	791
L-Kynurenine	HMDB00684	846
L-Lactic acid	HMDB00190	107689
L-Leucine	HMDB00687	6106
L-Lysine	HMDB00182	5962
L-Methionine	HMDB00696	6137
L-Phenylalanine	HMDB00159	6140
L-Proline	HMDB00162	145742
L-Serine	HMDB00187	5951
L-Threonine	HMDB00167	6288
L-Tryptophan	HMDB00929	6305
L-Tyrosine	HMDB00158	6057
L-Valine	HMDB00883	1182
Mandelic acid	HMDB00703	439616
Myoinositol	HMDB00211	892
N-Acetyl-b-glucosaminylamine	HMDB01104	897
N-Acetyl-D-mannosamine	HMDB11744	65150
N-Acetyl-L-aspartic acid	HMDB00812	65065
N-Acetylgalactosamine 4-sulphate	HMDB00781	11536759
N-Acetylglutamic acid	HMDB01138	185
N-Acetylneuraminic acid	HMDB00230	439197
N-acetylthreonine	NA	4651717
N-Acetylvaline	HMDB11757	227752
N-Methylnicotinamide	HMDB03152	64950
N1-Methyl-2-pyridone-5-carboxamide and others	HMDB04193	69698
Nicotinamide	HMDB01406	936
Nicotinic acid mononucleotide	HMDB01132	121991
O-Desmethylvenlafaxine glucuronide	NA	NA
O-Phosphoethanolamine	HMDB00224	1015
Orotic acid	HMDB00226	967
Oxoglutaric acid	HMDB00208	51
p-Cresol sulfate	HMDB11635	4615423
p-Hydroxymandelic acid	HMDB00822	NA
p-Hydroxyphenylacetic acid	HMDB00020	127
Pantetheine 4'-phosphate	HMDB01416	987
Pantothenic acid	HMDB00210	6613
Phenylsulfate	NA	74426
Phosphate	HMDB01429	1061
Phosphoenolpyruvic acid	HMDB00263	1005
Pimelic acid	HMDB00857	385
Pimpinellin or tetrahydroxybenzophenone	NA	NA
Proline betaine	HMDB04827	115244
Prolyl-4-hydroxyproline	HMDB06695	3952518
Propionylcarnitine	HMDB00824	107738
Pseudouridine	HMDB00767	15047
Pyridoxine	HMDB00239	1054

**Metabolite**	**HMDB ID**	**PubChem ID**
Pyroglutamic acid	HMDB00267	7405
Pyroglutamine	NA	NA
Pyrophosphate	HMDB00250	1023
Pyruvic acid	HMDB00243	1060
Quinic acid	HMDB03072	6508
Riboflavin	HMDB00244	6759
S-Adenosylhomocysteine	HMDB00939	439155
S-Adenosylmethionine	HMDB01185	1079
Salicyluric acid	HMDB00840	10253
Scyllitol	HMDB06088	892
Sorbitol	HMDB00247	5780
Suberic acid	HMDB00893	10457
Symmetric dimethylarginine	HMDB03334	169148
Tartaric acid	HMDB00956	444305
Taurine	HMDB00251	1123
Thymine	HMDB00262	1135
Tiglylglycine	HMDB00959	6441567
Uracil	HMDB00300	1174
Urea	HMDB00294	1176
Ureidopropionic acid	HMDB00026	111
Uric acid	HMDB00289	1175
Uridine diphosphate glucuronic acid	HMDB00935	17473
Vanillylmandelic acid	HMDB00291	736172
Xanthine	HMDB00292	1188
Xanthurenic acid	HMDB00881	5699
