# Supplementary material for: Assessing the metabolic effects of prednisolone in healthy volunteers using urine metabolic profiling
Source: Genome Med. 2012 Nov 30;4(11):94. doi: 10.1186/gm395 (PMC4064315; doi:10.1186/gm395)
Supplement: Additional file 3 — Table S2: List of all metabolites significantly regulated in urine of healthy volunteers treated with prednisolone, in protocol 1 and 2. Data represents the mean ratio of metabolite level at day 1 or day 15 compared to day 0. *P <0.05, **P <0.01,***P <0.001 and q <0.05 compared to placebo group for protocol 1 using LMMs, and compared to day 0 for protocol 2, using paired t tests. [file gm395-S3.PDF]

Supplemental Table 2: List of all metabolites significantly regulated in urine of healthy volunteers treated with prednisolone, in protocol 1 and 2.

**Metabolite**			**Day 1**					**Day 15**			
			**Protocol 1**				**Protocol 2**	**Protocol 1**			
**Pathway**	**Name**	**HMDB ID**	**Placebo**	**7.5 mg**	**15 mg**	**30 mg**	**75 mg**	**Placebo**	**7.5 mg**	**15 mg**	**30 mg**
Lipid metabolism	L-carnitine	HMDB00062	1.44	1.88	2.23	8.96***	8.37**	1.14	0.72	0.77	1.15
	L-Acetylcarnitine	HMDB00201	1.49	2.43**	2.82***	7.11***	5.88***	0.95	0.75	0.96	1.11
	Propionylcarnitine	HMDB00824	1.43	2.98	2.91**	5.21***	5.00***	0.98	1.29	1.05	1.09
	Myoinositol	HMDB00211	0.93	1.14	1.57	3.23***	4.49**	0.95	0.89	1.26	1.81
	Scyllitol	HMDB06088	1.00	1.12	1.05	1.31	1.51**	1.12	1.01	1.04	1.13
Proteinogenic amino acids	L-Lysine	HMDB00182	0.84	1.00	1.57	2.14***	2.14*	0.94	0.82	1.20	1.32
	L-Cysteine	HMDB00574	0.92	1.05	1.16	1.31	1.99**	1.00	1.10	1.06	1.18
	L-alanine	HMDB00161	1.08	1.14	1.29*	1.61***	1.93***	1.04	1.05	1.26	1.57***
	L-Histidine	HMDB00177	1.02	1.08	1.49	2.39***	1.92**	1.16	0.83	1.34	2.29**
	L-Methionine	HMDB00696	1.08	1.34	1.21	1.56***	1.82**	1.04	1.00	1.08	1.09
	L-Threonine	HMDB00167	0.98	1.05	1.17	1.57***	1.77**	0.92	0.91	1.03*	1.45***
	L-Proline	HMDB00162	1.01	1.17	1.20	1.73***	1.71**	1.21	1.05	1.00	1.10
	L-Serine	HMDB00187	1.03	1.10	1.18	1.46***	1.66**	1.00	0.92	1.01	1.22*
	L-Leucine	HMDB00687	1.01	1.16	1.03	1.43***	1.60*	1.00	0.97	1.01	1.11
	L-Valine	HMDB00883	1.04	1.08	1.07	1.42***	1.50***	1.08	0.94	1.02	1.16
	L-Asparagine	HMDB00168	0.75	0.94	1.00	1.24***	1.50	0.86	1.11**	1.34**	1.62***
	L-Glutamine	HMDB00641	0.61	0.75	0.64	1.03	1.47	0.95	1.49**	4.07*	3.30***
	L-Phenylalanine	HMDB00159	1.03	1.16	1.13	1.30***	1.45***	1.02	0.99	1.04	1.08
	Glycine	HMDB00123	0.98	0.99	1.22	1.37**	1.42*	0.96	0.90	1.08	1.21
	L-Isoleucine	HMDB00172	1.11	1.13	1.00	1.56**	1.38	1.22	0.95	0.87**	1.30
	L-Glutamic acid	HMDB00148	0.99	1.01	1.09	1.22	1.33**	1.13	1.01	1.03	0.97
	L-Tyrosine	HMDB00158	1.05	0.95	1.01	1.11	1.19*	1.03	0.95	1.04	1.15
	L-Tryptophan	HMDB00929	1.03	1.08	1.03	1.10	1.05	0.99	1.06	1.12	1.27***
Other amino acids	4-Guanidinobutanoic acid	HMDB03464	1.16	1.38	1.76	2.75***	4.00***	1.03	1.03	1.14	1.17
	Beta-Alanine	HMDB00056	1.02	1.37*	1.71*	2.17***	2.76*	0.96	0.72	0.82	0.78
	Pyroglutamine	NA	1.29	1.37	1.38	1.88**	1.94*	1.17	0.90	1.18	1.21
	3-Hydroxyisovaleric acid	HMDB00754	1.09	1.34	1.13	1.28	1.79*	1.08	1.22	0.96	1.11
	3-Methylhistidine	HMDB00479	1.42	1.40	1.23	1.45	1.71*	1.65	1.29	0.89	1.04
	Prolyl-4-hydroxyproline	HMDB06695	1.07	1.27	1.01	0.99	1.38	1.10	1.07	0.78**	0.86**

Metabolite			Day 1					Day 15			
			Protocol 1				Protocol 2	Protocol 1			
**Pathway**	**Name**	**HMDB ID**	**Placebo**	**7.5 mg**	**15 mg**	**30 mg**	**75 mg**	**Placebo**	**7.5 mg**	**15 mg**	**30 mg**
Other amino acids	L-Kynurenine	HMDB00684	1.06	1.11	1.58	1.44**	1.30*	0.98	1.03	1.12	1.20
	Kynurenic acid	HMDB00715	1.11	1.13	1.05	1.01	1.24	1.10	0.95	0.81***	0.87**
	gly-leu, acetyl-lys, ala-val	NA	1.08	1.10	1.07	1.16	1.18**	1.09	1.06	1.00	1.06
	Symmetric dimethylarginine	HMDB03334	1.06	1.15	1.02	1.09	1.10*	1.07	1.11	0.93	0.92
Carbohydrate metabolism	D-Glucose	HMDB00122	1.18	1.37	1.49	8.93***	16.72*	1.24	1.38	1.43	5.54*
	(S)-3-Hydroxyisobutyrate	HMDB00023	1.00	1.26	1.37*	1.96***	2.79**	1.01	1.16	1.26	1.38
	L-Lactic acid	HMDB00190	0.98	0.95	1.24	1.40	2.27**	1.12	1.03	1.42	1.54
	D-Xylitol	HMDB02917	1.10	1.15	1.08	1.08	1.18**	0.92	0.85	0.79	0.79
	Gluconic acid	HMDB00625	0.97	1.14	1.18	1.02	1.15	1.34	1.08	1.01**	0.89***
	Sorbitol	HMDB00247	0.94	1.02	0.84	0.85	1.02	1.20	0.97	0.90*	0.85*
	N-Acetylgalactosamine sulfate	HMDB00781	0.96	1.08	1.05	0.92	0.91	1.19	0.96	0.91	0.78***
Steroids	DHEA sulfate	HMDB01032	1.32	0.79	0.83*	0.80*	0.45*	0.95	0.23**	0.21**	0.22***
Energy metabolism	Citric acid	HMDB00094	1.06	1.06	1.20	1.23	1.33**	1.41	0.99*	0.96**	0.73***
	Isocitric acid	HMDB00193	1.11	1.22	1.20	1.17	1.14	1.13	1.09	0.91*	0.78***
	Phosphate	HMDB01429	1.25	1.31	0.93**	0.89**	0.91	1.07	1.03	0.81*	0.80**
Nucleotide metabolism	Guanine	HMDB00132	1.00	1.24	1.83	1.64	2.48*	1.67	1.11	2.12	1.70
	Thymine	HMDB00262	1.56	1.28	1.30	1.06	1.85*	1.14	1.45	1.49	1.18
	Nicotinic acid mononucleotide	HMDB01132	1.19	1.37	1.55**	1.31	1.42**	1.43	1.29	0.97*	0.94*
	Nicotinamide	HMDB01406	1.52	1.48	0.99**	0.99**	1.38	1.09	0.91	0.83	0.85
	Dihydrouracil	HMDB00076	0.99	1.37	1.53	1.40	1.34*	0.93	0.90	0.99	1.33
	Hypoxanthine	HMDB00157	1.10	1.20	1.19	1.17	1.22*	1.09	1.11	0.99	1.06
	(S)-b-aminoisobutyric acid	HMDB02166	1.18	1.25	1.09	1.11	1.21*	1.22	1.05	1.07	0.90
	Pseudouridine	HMDB00767	1.05	1.20	1.05	1.00	1.10	1.09	1.02	0.94	0.88*
	1-Methylguanosine	HMDB01563	1.03	1.16*	1.06	1.03	1.00	1.06	1.03	1.01	0.95
	N-Methylnicotinamide	HMDB03152	1.06	1.24	0.97	0.99	0.98	1.24	0.95	0.61*	0.48**
	5-methyluridine	HMDB00884	1.02	1.13	0.91	1.15	0.70**	1.42	1.25	0.92	1.18
Vitamins and cofactors	Pantothenic acid	HMDB00210	1.06	1.28	1.30*	1.33**	1.36**	0.92	0.96	1.07	0.90
	Pyridoxine	HMDB00239	1.25	1.13	1.01	0.97	1.34	1.24	0.66**	0.74	0.75**
	L-Dehydroascorbate	HMDB01264	1.08	1.19	1.08	1.23**	1.21	1.04	1.04	1.03	1.02
	4-Pyridoxic acid	HMDB00017	1.07	1.06	1.32	0.92	0.85*	1.06	0.99	1.36	0.86
Xenobiotics	Salicyluric acid	HMDB00840	1.13	1.28	0.81	0.94	1.60**	2.55	1.28	1.02**	0.95

Metabolite			Day 1					Day 15			
			Protocol 1				Protocol 2	Protocol 1			
**Pathway**	**Name**	**HMDB ID**	**Placebo**	**7.5 mg**	**15 mg**	**30 mg**	**75 mg**	**Placebo**	**7.5 mg**	**15 mg**	**30 mg**
Xenobiotics	p-Hydroxymandelic acid	HMDB00822	1.07	1.13	1.10	1.08	1.23*	1.03	1.07	1.05	1.11
	Tartaric acid	HMDB00956	1.08	1.16	1.03	0.97	0.91	1.10	1.12	0.88*	0.90
	Mandelic acid	HMDB00703	0.95	0.93	1.06	1.36**	0.89	1.01	1.07	0.96	1.46**
	Hippuric acid	HMDB00714	0.77	0.68	0.61	0.74	0.67**	1.14	1.09	0.86	0.88
Catecholamine metabolism	Dopamine	HMDB00073	1.12	1.15	1.05	0.97	1.22	1.27	0.93**	0.85***	0.88**
	Vanillylmandelic acid	HMDB00291	1.07	1.12	1.03	0.98	1.07	1.09	0.97	0.90	0.91*
	Homovanillic acid	HMDB00118	0.85	1.02	0.89	0.90	1.06	1.18	0.93*	0.88**	0.98*
Glycerophospholipid metabolism	Ethanolamine	HMDB00149	1.04	1.12	0.94	0.96	1.03	1.03	0.84**	0.80**	0.87*

Data represents the mean ratio of metabolite level at day 1 or day 15 compared to day 0.

*: $p<0.05$, **: $p<0.01$,***: $p<0.001$ and $q<0.05$ compared to placebo group for protocol 1 using LMMs, and compared to day 0 for protocol 2, using paired t-tests.
